# Deep dissection of stemness-related hierarchies in hepatocellular carcinoma

**DOI:** 10.1186/s12967-023-04425-8

**Published:** 2023-09-16

**Authors:** Rui Liang, Weifeng Hong, Yang Zhang, Di Ma, Jinwei Li, Yisong Shi, Qing Luo, Shisuo Du, Guanbin Song

**Affiliations:** 1 College of Bioengineering, Chongqing University, Key Laboratory of Biorheological Science and Technology, Ministry of Education, Chongqing, 400030 China; 2 Department of Radiation Oncology, Zhongshan Hospital, Fudan University, Xuhui District, No. 180, Fenglin Road, Xuhui District, Shanghai, China; 3 General Surgery 1, the First Affiliated Hospital of Dali University, Dali, 671000 China; 4 Department of Neurosurgery, The Fourth Affiliated Hospital of Guangxi Medical University, Liuzhou, 545000 Guangxi China

**Keywords:** Hepatocellular carcinomas, Stemness, Molecule subtypes, Prognosis, Tumor microenvironment, Immunotherapy, Immune infiltration, mRNAsi, scRNA-seq, LPCAT1

## Abstract

**Background:**

Increasing evidence suggests that hepatocellular carcinoma (HCC) stem cells (LCSCs) play an essential part in HCC recurrence, metastasis, and chemotherapy and radiotherapy resistance. Multiple studies have demonstrated that stemness-related genes facilitate the progression of tumors. However, the mechanism by which stemness-related genes contribute to HCC is not well understood. Here, we aim to construct a stemness-related score (SRscores) model for deeper analysis of stemness-related genes, assisting with the prognosis and individualized treatment of HCC patients.Further, we found that the gene LPCAT1 was highly expressed in tumor tissues by immunohistochemistry, and sphere-forming assay revealed that knockdown of LPCAT1 inhibited the sphere-forming ability of hepatocellular carcinoma cells.

**Methods:**

We used the TCGA-LIHC dataset to screen stemness-related genes of HCC from the MSigDB database. Prognosis, tumor microenvironment, immunological checkpoints, tumor immune dysfunction, rejection, treatment sensitivity, and putative biological pathways were examined. Random forest created the SRscores model. The anti-PD-1/anti-CTLA4 immunotherapy, tumor mutational burden, medication sensitivity, and cancer stem cell index were compared between the high- and low-risk score groups. We also examined risk scores for different cell types using single-cell RNA sequencing data and correlated transcription factor activity in cancer stem cells with SRscores genes. Finally, we tested core marker expression and biological functions.

**Results:**

Patients can be divided into two subtypes (Cluster1 and Cluster2) based on the TCGA-LIHC dataset's identification of 11 stemness-related genes. Additionally, a SRscores was developed based on subtypes. Cluster2 and the group with the lowest SRscores had superior survival and immunotherapy response than Cluster1 and the group with the highest SRscores. The group with a high SRscores was significantly more enriched in classical tumor pathways than the group with a low SRscores. Multiple transcription factors and SRscores genes are correlated. The core gene LPCAT1 is highly expressed in rat liver cancer tissues and promotes tumor cell sphere formation.

**Conclusion:**

A SRscores model can be utilized to predict the prognosis of HCC patients as well as their response to immunotherapy.

**Supplementary Information:**

The online version contains supplementary material available at 10.1186/s12967-023-04425-8.

## Introduction

Hepatocellular carcinoma (HCC) is the most predominant type of primary liver malignancy, accounting for approximately 90% of all prior liver cancer cases, and has become a significant public health problem [1]. HCC patients have the sixth-highest incidence and the fourth-highest mortality rate, according to global oncology statistics [2]. While morbidity and mortality rates are declining for many tumors, HCC incidence and mortality rates have increased significantly in many parts of the world, with a 43% increase in mortality from HCC in the United States between 2000 and 2016 [3, 4]. The majority of patients with HCC are diagnosed and treated at a late stage, with limited treatment options and a median survival of less than one year [5]. Although surgery, radiotherapy, and immunotherapy have helped improve morbidity and mortality in HCC, the overall 5 year survival rate is only 18% [4, 6]. Therefore, further research and exploration of the molecular mechanisms underlying the occurrence and development of HCC and the search for specific and sensitive biomarkers are essential for the early diagnosis, prognostic assessment, and advancement of effective treatment strategies for HCC.

Cancer stem cells (CSCs) are a subpopulation of cells in tumors that are in a stem cell-like state and have a unique ability to self-renew and differentiate [7, 8]. There is growing evidence for the presence of tumor cells with stem cell properties (liver cancer stem cells, LCSCs) in HCC. Liver stem/progenitor cells are transformed into LCSCs during long-term inflammatory processes induced by factors such as chronic viral infection or alcohol [9–11]. The study suggests that CSCs are primarily responsible for recurrence, metastasis, chemo, and radiation resistance in liver cancer [9, 12]. Therefore, targeting LCSCs therapy may become a strategy for treating HCC. Current single-cell transcriptomic analysis revealed that LCSCs exhibit heterogeneity, as well as that distinct genes in distinct subpopulations are independently associated with liver cancer prognosis, suggesting that additional LCSCs influence tumor progression and intra-tumor heterogeneity [13, 14].

CSC growth depends on their microenvironment. CSCs have the ability to interact with surrounding cells, release substances, and rearrange the nearby microenvironment to establish their own environment [15]. The CSC microenvironment contains cancer-associated fibroblasts (CAFs), endothelial cells, immunological cells, mesenchymal stem cells, and their growth factors and cytokines [15]. CSCs thrive in the complicated tumor microenvironment [15, 16]. CAFs promote CSC proliferation, invasion, and metastasis by secreting cytokine CXCL12, vascular endothelial growth factor, and stem cell growth factor [17]. They also support CSCs mechanically by producing fibrillar collagen. CSC microenvironments rely on endothelial cells. Hypoxia and vascular endothelial factors stimulate endothelial cell blood vessel growth. These new blood arteries feed CSC metabolism for self-renewal, invasion, and metastasis [18]. Endothelial cells release IL-1, IL-3, IL-6, VEGF-A, and other cytokines that support CSC proliferation and tumor growth [19]. Immune cells help CSCs invade and metastasize, attract T regulatory cells through TGF-β and cytokines, and elude the immune system [20]. Thus, knowing stemness-related genes and TME interactions in HCC helps develop tailored CSC therapeutics.

We examined stemness-related gene expression and prognosis in TCGA-LIHC patients screened for somatic mutations. Unsupervised clustering was used to classify hepatocellular cancer patients into two subgroups based on screening stemness-associated genes. Cox regression and random forest analysis were used to create stemness-related risk ratings from subtype-specific differentially expressed genes. Hepatocellular carcinoma subtypes and stemness-related risk scores were correlated with TME, drug sensitivity, chemotherapy/immunotherapy efficacy, and molecular function. We employed single-cell analysis to measure gene expression for stemness-related risk scores in distinct cells, and we annotated cancer stem cells to see their gene distribution. Intriguingly, we also examined transcription factors with variable expression in cancer stem cells and SRscores genes. Finally, LPCAT1, a stemness-related risk score core gene, was experimentally confirmed. The stemness-related sub-risk score predicted HCC patients' prognoses and guided treatment.

## Methods

### Raw data source and processing

The TCHA-LIHC cohort was downloaded from The Cancer Genome Atlas (TCGA, https://portal.gdc.cancer.gov/) and included 50 hepatocellular carcinoma paraneoplastic samples and 369 hepatocellular carcinoma samples [21]. Download ICGC-LIRI-JP from the International Cancer Genome Consortium (ICGC, https://dcc.icgc.org/), including 240 hepatocellular carcinoma samples [22]. Download GSE125449 and GSE151530 from the Gene Expression Omnibus database (GEO, https://www.ncbi.nlm.nih.gov/geo/), including 65 hepatocellular carcinoma samples [23, 24]. Twenty-nine stemness-associated gene sets were collected from the Molecular Marker Database (MSigDB, https://www.gsea-msigdb.org/gsea/msigdb/) (Additional file 4: Table S1) [25, 26], and 345 genes were included in the follow-up analysis after removal of duplicate genes (Additional file 5: Table S2).

### Based on multi-omics analysis

Differential expression of stemness-related genes in tumor and paraneoplastic tissues was analyzed by DESeq2 package in R, and adjP < 0.05 and |Log fold change |> 1 were used as screening thresholds [27]. One-way Cox regression analysis was performed using SurvivalR for all genes in the TCHA-LIHC cohort to identify genes significantly associated with Overall survival (OS). The intersection of differentially expressed stemness-associated genes and prognostic genes was determined using the VennDiagram package, and a Venn diagram was drawn. Mutations in stemness-related genes were analyzed using the maftools package to screen for genes with a mutation percentage more significant than 0 [28]. These genes' copy number variation (CNV) status was described using GISTIC 2.0, and chromosomal information and loss status were obtained and visualized by circos plot [29].

### Identification of stemness-related subtypes

Unsupervised clustering was performed in the TCGA-LIHC cohort via the Consensusclusterplus package based on genes with a proportion of mutations greater than 0 [30]. The K-means (km) cluster method with Euclidean distance was used in this analysis and repeated 1000 times to ensure reliability. And the effect of subtyping was examined by the PCA method, and Kaplan Meier survival curves were plotted for different subtypes using the survival package. The validation was performed in ICGC-LIRI-JP. To further explore the role of different subtypes in HCC, we used the GSVA package to compare potential action pathways between different subtypes. The CIBERSORT algorithm was used to compare the differences in immune cell infiltration between different subtypes. The ESTIMATE algorithm was used to compare the various subtypes of tumor microenvironments (TME). Also, we analyzed the expression of immune checkpoints between different subtypes.

### Prediction of chemotherapy sensitivity and response to immunotherapy

We assessed the efficacy response to multiple drugs in different stemness-related subtypes. First, the sensitivity of different subtypes to chemotherapeutic drugs (expressed as half-maximal inhibitory concentration IC50 values) was predicted by the pRRophetic package. In addition, we used the Tumor Immune Dysfunction and Exclusion (TIDE) online algorithm (http:// tide. dfci. harvard. edu/) to estimate the immunotherapeutic response in each HCC patient. Second, we downloaded the IPS scores of CTLA4 and PD1 from TCIA (https://tcia.at/home) for HCC patients and compared the effect of immunotherapy for different subtypes. [31]. Also, we compared. Finally, the sensitivity of stemness-related genes to non-immunotherapeutic drugs was observed using the GSCA database (http://bioinfo.life.hust.edu.cn/GSCA/#/) [32].

### Construction and validation of a prognostic stemness-related model

We further analyzed differentially expressed genes (DEGs) between the two subtypes in the TCHA-LIHC cohort to fully explore the stemness-related subtypes. And a one-way cox analysis (p < 0.05) was performed for the use of DEGs, a random forest model was built using the randomForestSRC package, and the genes ranked in the top 10 relative importance were combined by a permutation to determine the optimal signature: $Stemness Risk Score= {\sum }_{i=1}^{n}{Coef}_{i}\times {x}_{i}$, Where n represents the number of genes built by the scoring model, Coef equals the coefficient of each gene in the multifactorial cox regression, and x equals the expression of each stemness-related gene. SRscores = (0.075256372* expression of LPCAT1) + (−  0.004373688* expression of NDRG1) + (0.068014774* expression of G6PD) + (−  0.035236454* expression of CYP7A1) + (−  0.022288854* expression of DNASE1L3) + (0.051083710* expression of SPP1) + (0.004196020* expression of SFN) + (0.264500258* expression of CDCA8)..And to validate the prognostic value of the SRscores and the assessment of the accuracy of survival prediction. The regression coefficients in TCGA were then applied to the ICGC-LIRI-JP validation cohort to calculate the SRscores.

### Messenger RNA expression‑based stemness index (mRNAsi) calculation

Transcript mRNAsi values (ranging from 0 to 1) were directly calculated for each HCC sample by the TCGAbiolinks package (R version 4.2.0), strongly correlated with stem cell characteristics, and widely used for predicting tumor stemness. In the TCHA-LIHC cohort, the prognostic value of the mRNAsi index and its correlation with stemness-related subtypes and SRscores were further analyzed.

### Single cell analysis

Single-cell data of hepatocellular carcinoma were obtained from the publicly available dataset GEO (GSE125499 and GSE151530). The 10 × Genomic platform sequenced both datasets. For analysis using the R package Seurat, we filtered cells with UMI counts less than 200, while mitochondrial genes > 20% will also be filtered. The integrated data were screened for high variant genes, while the high variant genes were region-centric using the ScaleData function. The data are then subjected to PCA analysis and clustering analysis using the FindNeighbors process. Afterward, cell type identification was performed using the SingleR package with the reference gene HumanPrimaryCellAtlasData. In addition, we looked for markers of hepatocellular carcinoma stem cells (CD44, EPCAM, HAPLN1, HNF1B, IGFBP5, IHH, KRT19, LFNG, LGR5, NANOG, POU5F1, PROM1, SOX2, THY1) by CellMarker 2.0 and annotated the cells accordingly [33]. Stemness-related gene sets were scored in the single-cell dataset using the AddModuleScore function [34].

We analyzed all subpopulations of hepatocellular carcinoma single cells by R package SCENIC, inferred co-expression modules between transcription factors and candidate target genes based on the GENIE3 algorithm, and also performed cis-regulatory motif analysis (cisregulatory motif) for each co-expression module using RcisTarget [35]. Finally, the transcription factor activity was obtained by scoring each regulon activity of each cell using the AUCell algorithm, and the regulons were clustered by transcription factor activity [36]. The clustering method was used to calculate the correlation between transcription factor activities, to calculate the Connection Specificity Index (CSI) matrix of regulons, and to perform clustering, and the clustered modules were visualized in a UMAP plot. Finally, the average regulatory activity of each transcription factor was calculated and visualized.

### HE and immunohistochemistry

Paraffin-embedded tumor tissue was cut into 4 μm sections, dewaxed, and rehydrated. HE and immunohistochemical staining were performed according to standard protocols. HE staining: Sections were deparaffinized, stained with hematoxylin–eosin, dehydrated, transparent, and sealed, then photographed under microscopic (Nikon, ECLIPSE Ts2-FL) observation. Immunohistochemical staining: After slicing, dewaxing and antigen repair were performed, and the normal sheep serum working solution was sealed. Each tissue was dripped with LPCAT1 Polyclonal Antibody (protentech, 16112-1-AP) and incubated overnight at 4 ℃ in a refrigerator. Incubate the secondary antibody for 30 min after cleaning. Then, perform color rendering, re-staining, dehydration, and sealing. Finally, take photos using a microscope (Nikon, ECLIPSE Ts2-FL).

### Cell culture, shRNA transfection, and RTqPCR

The human HCC cell line HCCLM3 was purchased at Cellcook (https://www.cellcook.com/) and provided for genotyping identification.The HCCLM3 was cultured in DMEM (Gibco, C11995500BT) containing 10% (v/v) fetal bovine serum (FBS, ExCell Bio, FSP500) and 1% penicillin–streptomycin (penicillin 100 U/ml and streptomycin 0.1 mg/ml, Beyotime). And the cells were incubated at 37 °C in a 5% CO_2_ incubator. The shRNA against LPCAT1 and negative control (NC) were ordered in Beijing Tsingke Biotech (https://tsingke.com.cn/). The sequence of the shRNA is as follows: shLPCAT1-1( sense 5′–3′): CCGGTACCCGGATCAGACACATTTCTCTCGAGAGAAATGTGTCTGATCCGGGTATTTTTT; shLPCAT1-2( sense 5′–3′): CCGGAGATAGGTATTGCGGAGTTTGTCTCGAGACAAACTCCGCAATACCTATCTTTTTTT; shLPCAT1-3( sense 5′–3′): CCGGACGGAAAGTGGCCACAGATAATCTCGAGATTATCTGTGGCCACTTTCCGTTTTTTT.HCCLM3 was first spread into 6-well plates, and after growing to 70–80%,the cells were transfected with plasmids by polyethylenimine (PEI, Polysciences, 23966–1). RTqPCR detected the knockdown efficiency of HCCLM3. Total RNA was extracted with TRIzol reagent (Takara) and then reversed and transcribed into cDNA using the PrimeScript RT Master Mix kit (Takara, RR036A). RT-PCR was performed using TB Green^®^ Premix Ex Taq™ II (Takara,RR820A). RT-qPCR detected relative expression of LPCAT1. GAPDH was used as a reference for endogenous normalization. The sequence of LPCAT1: (Forward) TATTCCGAGCCATTGACCAAGAG, (Reverse) GAAATGTGTCTGATCCGGGTACA.The sequence of GAPDH: (Forward) GGTATGACAACGAATTTGGC, (Reverse) GAGCACAGGGTACTTTATTG.

### Tumorsphere formation assay

HCCLM3 cells (5 × 10^3^ cells/well) were inoculated into ultra-low adherence well plates with 2 ml of DMEM-F12 medium (Gibco,C11330500BT) supplemented with bFGF (Peprotech, 100-18B-10), EGF (Peprotech,AF-100–15), N2(Gibco, 17,502,048), and B27 cytokines (Gibco, 17,504,044). The tumor spheroids were observed and photographed under the microscope (LEICA,DMI3000 B) after 4–5 days of culture.

### Statistical analysis

All statistics were performed using R3.6.3 and R4.2.0 versions. The Wilcoxon test (Wilcoxon rank sum test) was used for two sets of continuous type variable data. The Spearman correlation test was used for the correlation between gene expressions. Univariate and multifactorial Cox regression was used to define prognostic correlates, ROC curves were calculated using the timeROC package, and the area under the line was used to assess the prognostic effect of the SRscores. p < 0.05 was used as a criterion for the statistical significance of differences.

## Results

### Expression and mutation analysis of stemness-related genes in HCC

**Fig. 1:**
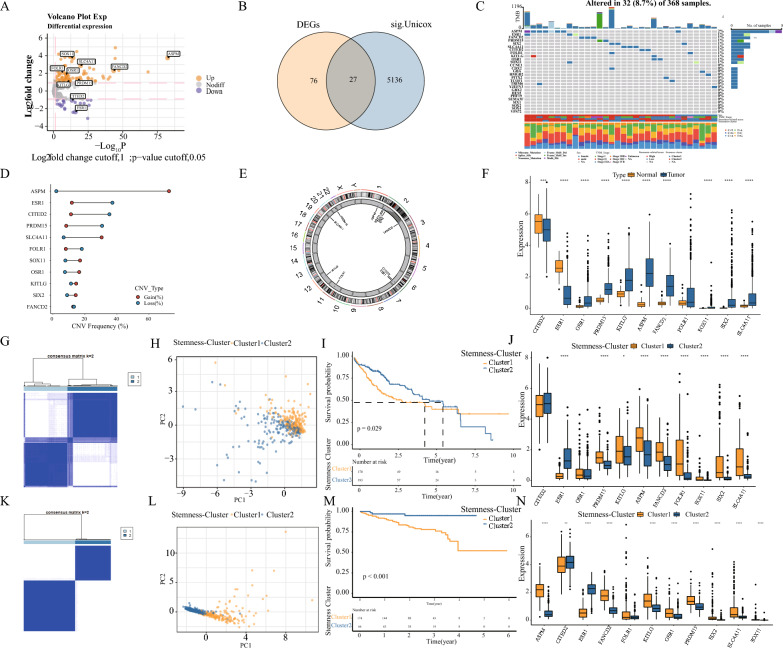
Identification of stemness-related genes and subtypes in HCC. **A** Volcano map showing 103 of 345 stemness-associated genes with differential mRNA expression in HCC data. **B** Venn diagram showing 27 stemness-related genes with differential expression has prognostic value. **C** Waterfall plot showing the mutational landscape of 27 stemness-associated genes. **D** Copy number variation (CNV) of 11 stemness-associated genes indicates increased CNV and green dots indicate decreased CNV. **E** Position of CNV of 11 stemness-associated genes on the chromosome. **F** Differences in mRNA expression of 11 stemness-associated genes between HCC and normal tissues, *p < 0.05; **p < 0.01; ***p < 0. 001; ****p < 0.0001; ns, no statistical significance. **G** Identify two stemness-related subtypes by unsupervised clustering. **H** PCA shows the difference between the two subtypes. **I** Kaplan–Meier curves show survival differences between the two subtypes. **J** Differential expression of 11 stemness-associated genes in the two subtypes. **K**–**N** Validation in ICGC-LIRI-JP data

The data from TCGA-LIHC were analyzed comprehensively. Differential analysis between liver cancer and paracancer tissues revealed differences in 103 out of 345 stemness-related genes, with up-regulated and down-regulated genes as shown in Fig. 1A volcano plot. A univariate Cox analysis identified 5163 prognosis-associated genes in the TCGA-LIHC. The intersection was taken with 103 stemness-associated genes, and 27 stemness-associated genes showed differential and prognostic values in HCC (Fig. 1B). In a one-way cox analysis of these 27 genes, two genes were found to be protective factors for HR < 1, and 22 genes were risk factors for HR > 1 (Additional file 1: Fig. S1A). To investigate the genetic mutation of these genes in HCC, we analyzed the incidence of somatic mutations and found that 11 of the 27 genes associated with stemness had a mutation frequency of > 1% (Fig. 1C). In addition, copy number variants (CNV) were analyzed, and copy number alterations were found to be prevalent in 11 stemness-associated genes (Fig. 1D). Among them, there were extensive CNV increases in ASPM, SLC4A11, SOX11, and OSR1, while CNV decreases in ESR1, CITED2, PRDM15, and FOLR1. Figure 1E shows the chromosomal locations of the 11 stemness-associated genes with CNV variants. Figure 1F demonstrates that the expression of these 11 genes at the mRNA level differs substantially between tumor and normal tissue. Consequently, stemness-related genes may play a crucial role in the development of HCC.

### Identification of subtypes of HCC by stemness-associated genes

Clustering analysis of 363 tumor patients with TCGA-LIHC based on 11 stemness-associated genes using the ConensusClusterPlus R package revealed two subtypes, including 168 cases in Cluster 1 and 187 cases in Cluster 2 (Fig. 1G). Additional file 1: Fig. S1B depicts the interactions of these 11 stemness-associated genes. Using PCA analysis to evaluate the differences between the two subtypes, it was determined that the two stemness subtypes differed markedly in the transcriptome (Fig. 1H). Survival analysis was also used to evaluate the prognostic value of the two subtypes of stemness in clinical practice. The OS of patients with both subtypes was significantly different (p = 0.029), with Cluster 1 having a significantly worse prognosis (Fig. 1I). Nine of these 11 stemness-related genes displayed differential expression between the two subtypes, as shown in Fig. 1J. To verify these two typings' stability and applicability, we validated unsupervised cluster analysis using ICGC-LIRI-JP. It was well divided into two categories (Fig. 1K), PCA analysis was significantly different (Fig. 1L), survival was quite different between the two subtypes (p = 0.00091). Cluster1 had a significantly poorer prognosis (Fig. 1M), and ten of eleven stemness-related genes were significantly different between the two subtypes (Fig. 1N). Using the TCGA-LIHC dataset, we analyzed the distribution of somatic mutations for high and low SRscores and found that TP53 was predominant in Cluster1 patients. At the same time, CTNNB1 predominated among patients in Cluster 2 (Additional file 1: Fig. S1C, D). Somatic mutations result from diverse mutational processes such as DNA repair defects and exposure to exogenous or endogenous mutagens. Different mutational processes produce different mutation types, i.e., mutational characteristics. Therefore we used NMF to characterize the genomic landscape comprehensively. In Cluster1, MMR and Guanine damage predominate, while in Cluster2, MMR and Adenine damage predominate (Additional file 1: Fig. S1E, F).

### Analysis of tumor microenvironment and signaling pathways between two subtypes in HCC

**Fig. 2:**
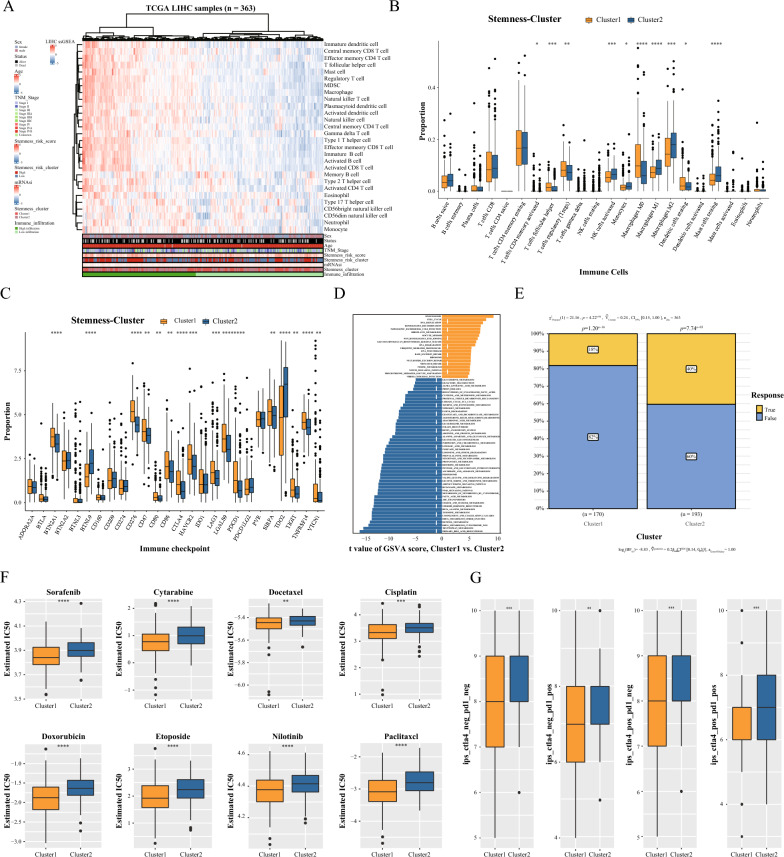
Tumor microenvironment, signaling pathway analysis, and drug sensitivity analysis among different subtypes. **A** Heat map showing the differences of different immune cell subpopulations in the two subtypes. **B** Relative abundance of each immune cell subpopulation between two subtypes. **C** Analysis of immune checkpoint expression in two subtypes. **D** Enrichment analysis of GSVA pathway between the two subtypes. **E** TIDE algorithm to estimate immunotherapy response in patients of both subtypes. **F** Box plot of estimated IC50 of chemotherapeutic drugs between two subtypes. **G** Analysis of immune checkpoint blockade therapy (e.g., anti-PD-1 and anti-CTLA4) between the two subtypes

By comparing the infiltrative immune cell component in the TME between the two subtypes, we discovered a higher rate of immune cell infiltration in Cluster1 in addition to the immune hyper-infiltrative zone depicted by the heat map (Fig. 2A). In the heat map, it was found that in the high immune infiltration area, anti-immune response cells such as Type 1 T helper cells, activated CD8 + T cells, and immunosuppressive cells such as immature dendritic cells, regulatory T cells, and neutrophils were significantly enriched. Interestingly, most of the samples in Cluster1 showed low immune infiltration, while most of the samples in Cluster2 showed high immune infiltration. These suggest that TME may promote the recruitment or differentiation of immune cells [37].To distinguish the specific immune components between the two subtypes in the tumor immune microenvironment, the distinctions between 28 immune cells were calculated (Fig. 2B). Due to the disparities in immune infiltration between the two subtypes, we further analyzed the differences between immune checkpoints. The results showed considerable immune checkpoint differences between the two subtypes (Fig. 2C). To further explore the possible mechanisms between the different subtypes, we analyzed the differences in enrichment pathways between the two subtypes based on GSVA. The results showed that Cluster1 was mainly enriched in cell cycle, DNA replication, homologous recombination, and notch signaling pathway, while Cluster2 has enriched primarily in glutathione metabolism, α-linolenic acid metabolism, Cysteine and methionine metabolism, or other metabolism-related pathways were mainly enriched in Cluster2 (Fig. 2D). Glutathione promotes T-cell activation, proliferation, and differentiation and is essential for maintaining T cell immunity [38].α-linolenic acid plays a protective role in various tumors.MDSCs may limit T cell activation by limiting the effectiveness of cysteine on T cells [39]. And methionine metabolism correlates with the number of CD8 + T cells [40]. These results suggest that these pathways play an essential role in the immune infiltration of tumors, but the specific complex mechanisms need to be explored by more in-depth experiments.

### Analysis of chemotherapy sensitivity and immunotherapeutic response between two subtypes in HCC

Chemotherapy remains the standard treatment for cancer patients. Using pRophetic algorithm, we determined the sensitivity of both subtypes to conventional chemotherapeutic medications.Cluster1 was found to be more sensitive to Sorafenib, Cytarabine, Cisplatin, Doxorubicin, and other medications (Fig. 2F). Using the TIDE algorithm, we also assessed the immunotherapeutic response of both subtypes. Figure 2E demonstrates that immunotherapy benefited more Cluster2 patients than Cluster1 patients. The effect of immunotherapy on the immune checkpoints PD-1 and CTLA4 in patients with both subtypes of TCIA was investigated further. Figure 2G demonstrates that Cluster2 was more efficacious against CTLA4, anti-PD-1, and combinations of both. These results indicate that stemness subtypes  is associated with immunotherapy and chemotherapy sensitivity.

### Construction and validation of a prognostic model based on the analysis of differences between two subtypes

**Fig. 3:**
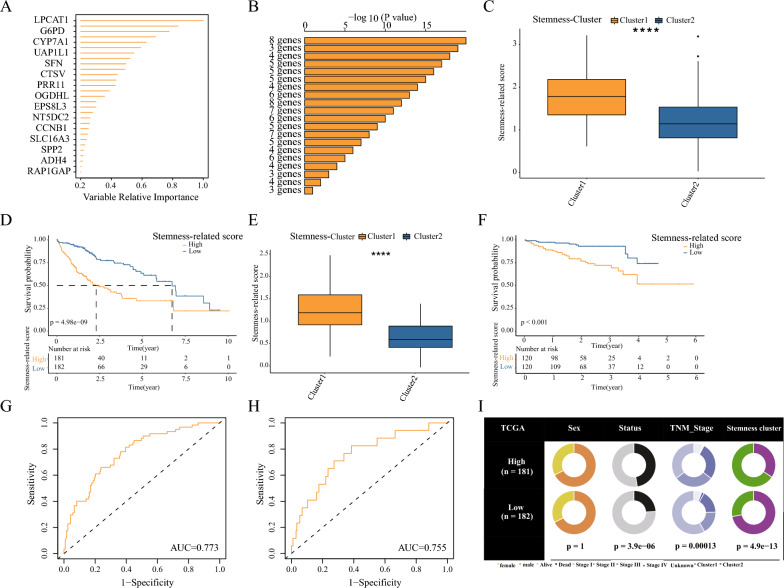
Identification of prognostic stemness-related signatures (**A**) Random forest algorithm to calculate the top genes' relative importance. **B** The top 10 genes were subjected to 1023 (2^10^–1) permutations and log-rank tests, and the top 8 genes were selected for model construction. **C** Analysis of SRscores among different subtypes in TCGA-LIHC data, ***p < 0. 001. **D** Analysis of survival differences between high and low SRscores groups in TCGA-LIHC data. **E**, **F** ICGC-LIRI-JP data to validate the fitness of the model. **G**, **H** Time-dependent ROC analysis showing the specificity of SRscores in TCGA-LIHC (training set) and ICGC-LIRI-JP (validation set). **I** Pie charts showing the cardinality test for clinicopathological factors and stemness subgroups for each group of the stemness score

First, we analyzed the DEGs between the two subtypes and obtained 550 genes. Then, random forest analysis filtered out the genes with high relative importance (Fig. 3A). Subsequently, a log-rank test was performed by combining these 10 genes in 1023 (2^10^–1) permutations, and the prognostic model was evaluated. Figure 3B shows the − log10 (log-rank P) values of the top 20 models, from which the highest ranking signature consists of 8 genes (LPCAT1, NDRG1, G6PD, CYP7A1, DNASE1L3, SPP1, SFN, CDCA8) was selected for the construction of the stemness risk model. The SRscores was calculated for each HCC patient by this score.

Figure 3C reveals that Cluster1 patients had a substantially higher SRscores (p < 0.001) than Cluster2 patients. Patients with a high SRscores had a substantially worse prognosis than those with a low score (p < 0.001) (Fig. 3D). In TCGA-LIHC, a time-dependent ROC curve analysis determined the sensitivity and specificity of the SRscores, with an AUC of 0.773% (Fig. 3G). ICGC-LIRI-JP was then validated in order to establish the model's dependability. Figure 3E reveals that Cluster1 patients had a higher SRscores than Cluster2 patients. In the survival analysis, patients with a high SRscores had a substantially worse prognosis (Fig. 3F), with a time-dependent ROC curve AUC value of 0.75 (Fig. 3H). The pie chart demonstrates that patients with higher risk scores have more advanced pathological staging than those with lower risk scores. (Fig. 3I). The aforementioned findings indicate that the risk model is highly adaptable and has an outstanding prognostic effect on HCC patients.

### Genomic characterization of high and low SRscores, TMB, tumor microenvironment, and signaling pathway analysis

**Fig. 4:**
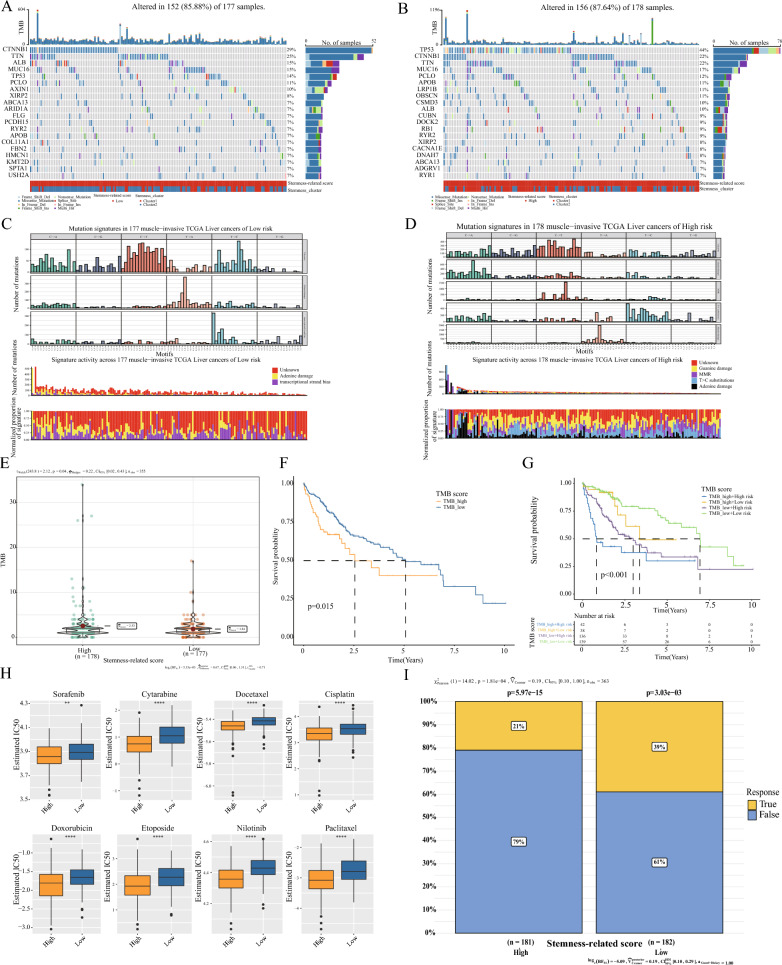
Mutation, TMB, and drug sensitivity analysis in SRscores groups. **A**, **B** In the SRscores group, waterfall plots show the distribution of somatic mutations in the genes with the highest mutation frequencies. **C**, **D** Bayesian NMF identification of mutation markers in the SRscores group. The middle and following plots show the relative proportions of the total number of mutations and mutation types. **E** Differences in TMB between high and low SRscores groups. **F** Prognostic analysis of the high TMB and low TMB groups. **G** Prognostic analysis of the combined interventional SRscores and TMB. **H** Box plot of estimated IC50 of chemotherapeutic drugs between SRscores group. **I** TIDE algorithm for immunotherapy response between the high-SRscores group and the low-SRscores group

Using the TCGA-LIHC dataset, we analyzed the distribution of somatic mutations in high and low SRscores. The analysis revealed that patients with low-risk scores were predominately CTNNB1 positive. Similarly, TP53 was prevalent among patients with high-risk scores (Fig. 4A, B). Additionally, we exhaustively characterized the genomic landscape. Figure 4C, D demonstrates that MMR and Guanine damage dominated the low-risk score, whereas MMR and Adenine damage were vanquished in the high-risk score. Calculating the TMB for each patient with HCC, we discovered that the TMB was greater in the group with a high stem risk score (Fig. 4E). By prognostic analysis, we found that the high TMB group had a poorer prognosis than the low TMB group (Fig. 4F). More importantly, by combining TMB grouping and SRscores for survival analysis, patients with low TMB and low SRscores had significantly longer OS than patients with high TMB and high SRscores (Fig. 4G). We also performed GSVA pathway enrichment analysis between high and low SRscores groups. The results showed that cell cycle,mTOR signaling pathway,P53 signaling pathway,NOTCH signaling pathway, and other pathways closely related to tumor progression were significantly enriched in the high-stem risk group. In contrast, in the low SRscores group, metabolism-related pathways such as FATTY_ACID_METABOLISM, RETINOL_METABOLISM, TYROSINE_METABOLISM, GLYCINE_SERINE_AND_THREONINE_METABOLISM were mainly enriched (Additional file 1: Fig. S1I). Such is also comparable to the subtype results. In addition, we calculated the immune cell subpopulation infiltration in the high and low SRscores groups, and found greater Eosinophil and Neutrophil infiltration in the low SRscores group, which was associated with improved survival, compared to the high SRscores group. (Additional file 2: Fig. S2A). We further analyzed the differences between immune checkpoints. The results showed considerable immune checkpoint differences between the high and low SRscores groups (Additional file 2: Fig. S2B). We further described the correlations of the eight genes that constitute the stemness risk model, and there was a significant negative correlation between most of them (Additional file 1: Fig. S1G, H). In addition, these genes had substantial correlations with multiple immune cells (Additional file 2: Fig. S2C).These results suggest that these genes may contribute to the biological differences between the high and low-scoring groups.

Additionally, we examined the expression of these eight genes in cancerous and paraneoplastic tissues using the TCGA-LIHC dataset. The expression of LPCAT1, NDRG1, G6PD, CYP7A1, SPP1, SFN, and CDCA8 was significantly higher in cancer tissues than in paraneoplastic tissues, whereas the expression of DNASE1L3 was significantly lower in cancer tissues (Additional file 1: Fig. S1J). We used the IOBR package to analyze the correlation between high and low SRscores groups and multiple tumor-related characteristics datasets. It was found that the high-stem risk score group was significantly enriched in classical tumor pathways such as WNT, TGF-β, JAK-STAT3, mTOR, etc. (Additional file 2: Fig. S2G). We also used the GSCA database to assess the correlation between genes of SRscores and pathways such as apoptosis, cell cycle, and EMT. We found that most genes were involved in the activation of pathways of tumor progression (Additional file 2: Fig. S2D).These results suggest these genes may contribute to tumor progression and poor prognosis.

### Analysis of pharmacotherapy for high and low SRscores

Using the GSCA database, we analyzed the sensitivity of genes and chemotherapeutic medications for the SRscores. Additional file 2: Fig. S2E, F demonstrates that SPP1, SFN, NDRG1, and G6PD are sensitive to the majority of medications. Using the pRophetic software, we also determined the sensitivity of high and low SRscores categories to chemotherapy drugs. Figure 4H demonstrates that the group with a high SRscores was more sensitive to Sorafenib, Cytarabine, Cisplatin, and Doxorubicin than the group with a low risk score. Similar to the subtypes, the group with a reduced risk score and a better prognosis benefited more from immunotherapy (Fig. 4I). We also investigated the effect of immunotherapy with the immune regulators PD-1 and CTLA4 on scores indicating high and low stemness risk. There was no significant difference between the two groups, unfortunately.These results can assist in selecting chemotherapeutic agents when immunotherapy is combined with chemotherapy in clinical practice.

### Analysis of mRNAsi binding subtypes and SRscores

**Fig. 5:**
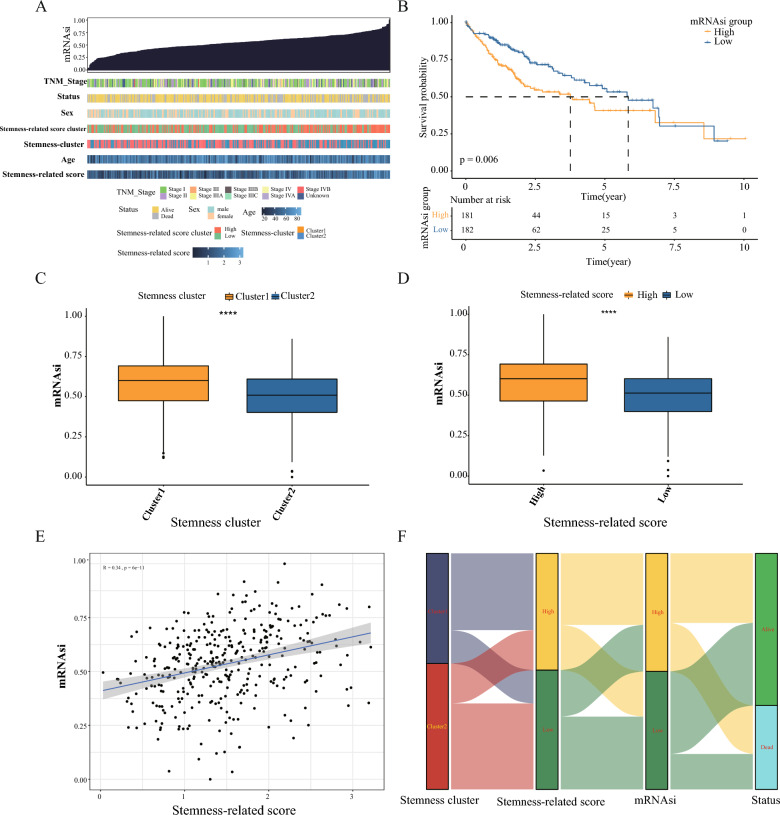
mRNAsi analysis and Single-cell analysis validation. **A** Correlation between mRNAsi and different subtypes, SRscores, and clinical features are shown. **B** Prognostic analysis of high mRNAsi group and low mRNAsi group. **C**, **D** Analysis of mRNAsi in different subtypes and SRscores groups, ***p < 0. 001. **E** Spearman correlation analysis of SRscores with mRNAsi. **F** Sankey plots show the relationship between different subtypes, high and low SRscores groups, high and low mRNAsi groups, and prognostic outcomes in HCC

The new version of the TCGAbiolinks package calculated the mRNAsi of each patient in the TCGA-LIHC dataset. We investigated the relationship between mRNAsi, stemness subtypes, and SRscores. We ranked HCC samples according to their mRNAsi values from low to high and examined their correlation with stemness subtypes, SRscores, and clinical characteristics (Fig. 5A). Cluster1 and the group with a high SRscores were found to be highly concentrated in the high mRNAsi region. In contrast, Cluster2 and the group with a low SRscores were primarily concentrated on the low mRNAsi region, as depicted in Fig. 5C, D.

mRNAsi and SRscores showed a significant positive correlation (R = 0.34, P < 0.001), as shown in Fig. 5E. In addition, the prognostic analysis demonstrated that patients with elevated mRNAsi had a worse prognosis than those with low mRNAsi (Fig. 5B). Figure 5F depicts the distribution of HCC patients between these three subtypes using a Sankey diagram, and the results are consistent with those described previously.The distribution of HCC patients between subtypes, SRscores groups, and mRNAsi groups was presented using the Sankey diagram. The results were also consistent with those described above, with samples from the better prognostic Cluster2, low SRscores groups, and low-mRNAsi groups essentially overlapping. Samples from the poorer prognostic Cluster1, high SRscores groups, and high-mRNAsi groups overlap (Fig. 5F).

### Single cell analysis of SRscores

**Fig. 6:**
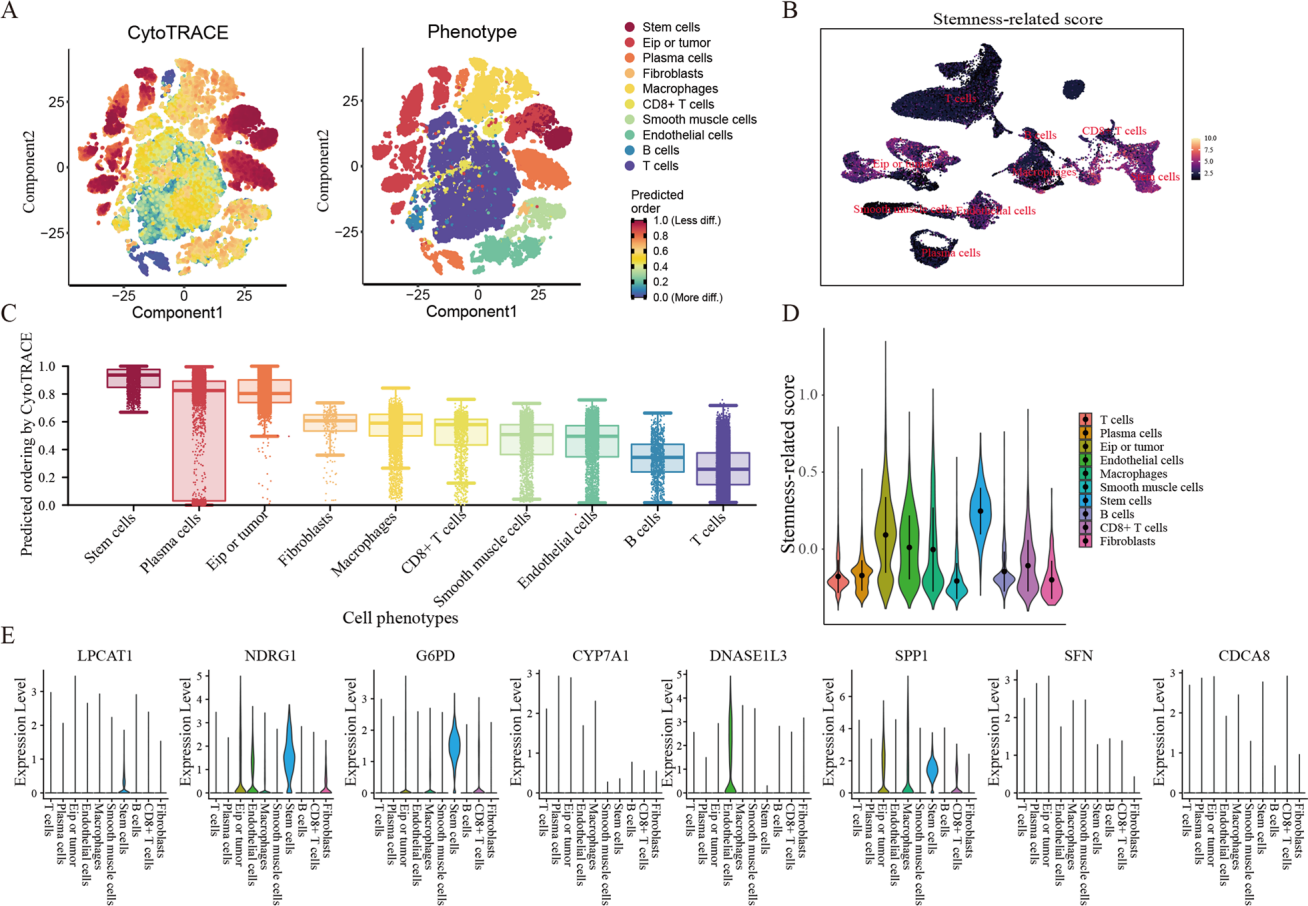
Single-cell annotations and SRscores in single cells. **A** t-distribution random neighborhood embedding (tSNE) plots of malignant cells from HCC. **B**, **D** Display of SRscores in each cell subpopulation. **C** CytoTRACE scores of each cell population. **E** Expression of the eight genes constituting the SRscores in each cell subpopulation

To further explore the expression profile of the SRscores, we analyzed its distribution and expression in the scRNA-seq dataset. First, we downloaded the single-cell dataset (GSE125449, GSE151530) from the GEO database with quality control (Additional file 3: Fig. S3A, C). Subsequently, the UMAP and tSNE algorithms were used to cluster all cells, which could be divided into 31 clusters (Additional file 3: Fig. S3B). According to marker genes, these 31 clusters were annotated as B cells, Endothelial cells, Plasma cells, Macrophages, Smooth muscle cells, stem cells, T cells, CD8 + T cells, and Fibroblasts (Additional file 3: Fig. S3D, E). To further validate the accuracy of cancer stem cell clustering, we also used the CytoTRACE package to calculate the stem cell characteristic scores (i.e. CytoTRACE scores) for each cell, with CytoTRACE scores ranging from 0 to 1, while higher scores indicate higher stemness (less differentiation) and vice versa. In turn, the tumor stemness level of each cell was assessed, with high scores corresponding to high tumor stemness (Fig. 6A). As expected, we found that the stem cells we clustered had significantly higher CytoTRACE scores compared to other cell populations (Fig. 6C) [41, 42]. In addition, we found that in the tumor tissues of HCC patients, high SRscores were shown in Hepatocytes (Fig. 6B, D, Additional file 3: Fig. S3F). Interestingly, we further analyzed the expression of eight genes of the stemness score risk model in these cells and found the highest expression in stem cells (Fig. 6E, Additional file 3: Fig. S3G). Finally, we analyzed the co-localization of LPCAT1 and NDRG1, the two genes with the most substantial importance shown by random forest, and found a strong localization consistency (Additional file 3: Fig. S3H).

**Fig. 7:**
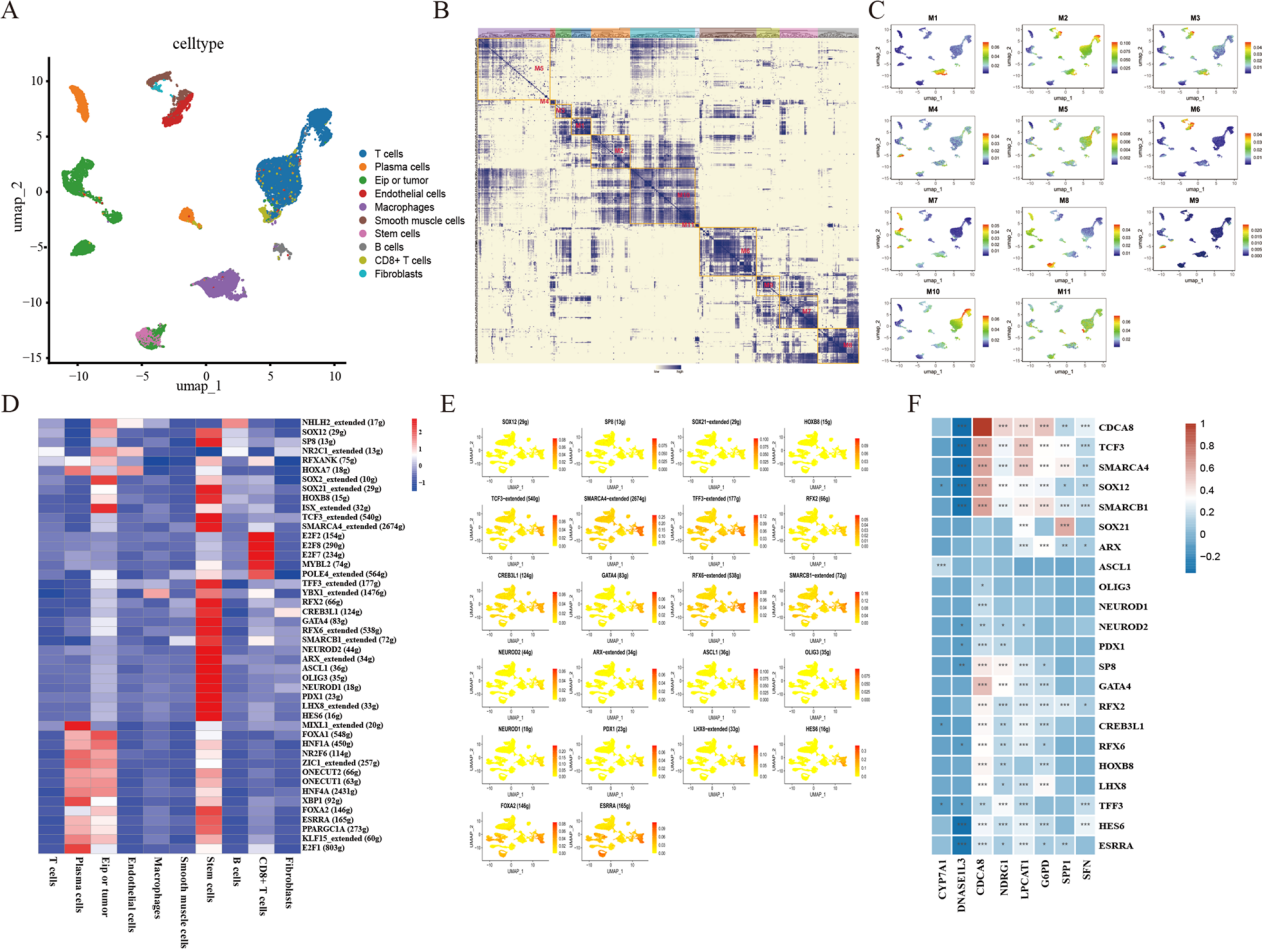
Correlation of transcription factors and stemness-related genes was confirmed by single-cell analysis. **A** Cell annotations for the HCC-integrated dataset are shown on the UMAP plots, with different colored representations of cell types. **B** Identify regulatory modules based on the regulator CSI matrix and extract core transcription factors, binding motifs, and corresponding cell types. **C** The distribution of transcription factors among cell populations in each module. **D** Heat map showing the expression of the screened transcription factors in each cell population. **E** The enrichment of the screened transcription factors in each cell population. **F** Heat map display of the correlation between screened transcription factors and stemness-related genes

It is well known that transcription factors play an important role in tumor development, and in addition, they play an indispensable role in the promotion of tumor development by tumor stem cells [43–46]. To investigate the expression activity of transcription factors in tumor stem cells and the correlation with SRscores genes, we performed a single-cell clustering analysis. First, we classified the cells within the liver cancer tissue into 11 cell clusters by transcription factor clustering effects and found that the stem cells were mainly in the M8 cluster (Fig. 7A–C). Furthermore, after calculating the average regulatory activity of transcription factors in the M8 module, we screened the transcription factors with an average regulatory activity > 2 in stem cells for visualization and correlation analysis with stemness genes and found that genes such as TCF3, SMARCA4, TFF3, RFX6, SMARCB1, and HES6 were enriched in stem cells (Fig. 7D, E). Finally, we analyzed the correlation between these transcription factors and stemness-related genes in hepatocellular carcinoma transcriptome data and found a significant correlation between NDRG1 and these transcription factors compared to LPCAT1 (Fig. 7F).

### LPCAT1 regulates the stemness of HCC

**Fig. 8:**
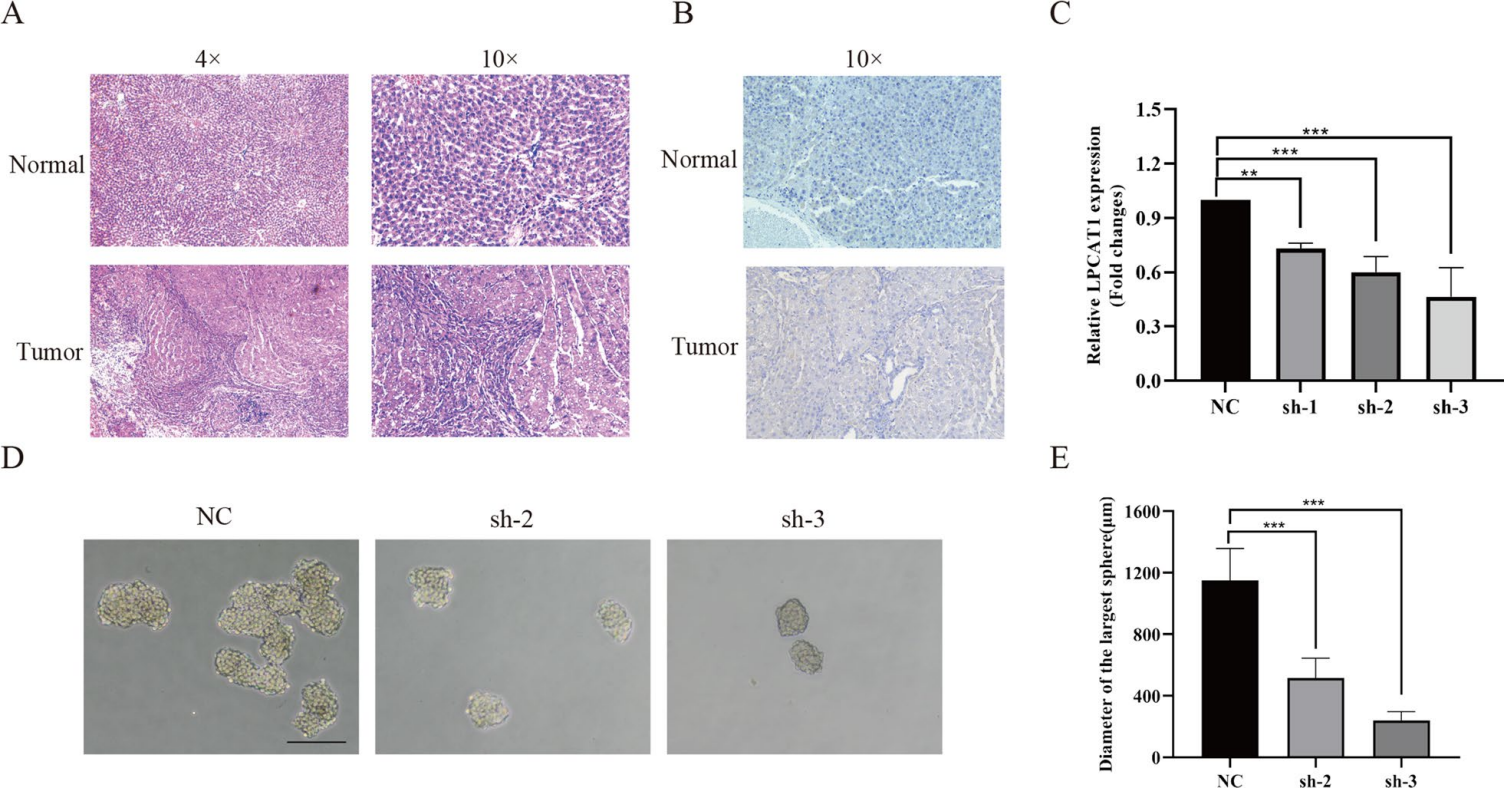
LPCAT1 expression and stemness in HCC (**A**) Hematoxylin–Eosin staining detection of normal and hepatocellular carcinoma (**B**) Immunohistochemical detection of LPCAT1 expression in normal and hepatocellular carcinoma groups. **C** Knockdown of LPCAT1 in HCCLM3 cells and validated by qRT-PCR, **p < 0.01;***p < 0. 001. **D**, **E** Sphere formation assay was performed after transfection of HCCLM3 cells using NC, sh-2, and sh-3, the sphere size of each group was photographed, and the maximum sphere diameter statistic was performed, Scale bar of 200 μm is shown, ***p < 0. 001

Random forest analysis revealed that LPCAT1 is the most critical gene in the stemness risk model. Therefore, we validated LPCAT1 expression and potential function in CSC characterization at a histological and cellular level. The basis of our previous work has established a batch of rat models of hepatocellular carcinoma with preserved paraffin specimens [47]. HE staining confirmed the successful construction of hepatocellular carcinoma (Fig. 8A), and immunohistochemistry results showed that the expression of LPCAT1 was significantly higher in HCC than in normal rat liver tissue (Fig. 8B). The study reported the highest expression of LPCAT1 in HCCLM3 in hepatocellular carcinoma cells in vitro [48]. Therefore, we selected specific shRNAs to inhibit LPCAT1 expression in HCCLM3 (shRNA-1, shRNA-2, shRNA-3; Fig. 8C). We then performed sphere formation experiments using the more efficient knockdown shRNA-2, shRNA-3, and the control group. We found that the maximum diameter of spheres was significantly reduced in the shRNA group compared to the NC group (Fig. 8D, E). It showed that the knockdown of LPCAT1 could inhibit the stemness characteristics of HCC cells.

## Discussion

Due to their distinct biological functions in tumors, CSCs have gained the interest of numerous researchers in recent years. Consequently, they play a vital role in the progression, metastasis, recurrence, and radiotherapy resistance of HCC [9, 12]. Growing evidence suggests that LCSCs are the primary organizers of HCC initiation, such as liver tumor initiating cells [49]. Nonetheless, the physiopathology and mechanisms of LCSCs in hepatocellular carcinoma require further investigation. Here, we analyzed the role of stemness-related genes in HCC using bioinformatics to examine the molecular characteristics of these genes. Moreover, the key resuts were validated in the experiements.We obtained 11 stemness-associated genes based on a multi-omics screen for unsupervised cluster analysis, identified two subtypes, and constructed a SRscores by subtypes. The differences in survival status, TME, somatic mutations, and drug sensitivity of HCC patients by subtyping and SRscores were evaluated. These studies provide a more accurate prognostic analysis for HCC patients.

HCC is difficult to diagnose in its early stages, and patients with advanced disease have a poor prognosis. With the advancement of immunotherapy, oncology is increasingly employing immunotherapeutic agents. In clinical treatment, immunodetection inhibitors such as anti-PD1, anti-PDL1, and anti-CTLA4 monoclonal antibody therapy are widely utilized. TME provides a suitable environment for protecting and regulating CSCs, which supports the growth and differentiation of CSCs and thus promotes tumor metastasis [50]. Understanding the characteristics of CSCs and TMEs in HCC is crucial. CSCs can secrete immunosuppressive cytokines that enable tumor-associated macrophages to secrete multiple inflammatory cytokines and recruit myeloid-derived suppressor cells (MDSCs), thereby facilitating the formation of a tumor microenvironment conducive to CSCs' survival [51, 52]. Our findings also validate that macrophages and MDSCs were more abundant in the high SRscores group. In addition, some biomarkers, such as PD-L1, human epidermal growth factor receptor 2 (HER2), vascular growth factor (VEGF), and TMB are highly predictive of tumor immunotherapy [53, 54]. We analyzed the efficacy of immunotherapy using data from TIDE and TCIA databases on HCC patients. We observed that patients in Cluster 2 and those with a low stemness score were more responsive to immunotherapy. Moreover, Cluster2 patients were more amenable to anti-PD-1 and anti-CTLA4 therapies.

Malta et al. developed the OCLR technique to calculate the transcriptional stem cell index (mRNAsi) using the 11,774-gene mRNA expression profile [55, 56]. We used the same method to calculate the mRNAsi of HCC patients. Our findings are similar to previous studies in that patients with high mRNAsi had a poorer prognosis [57]. Patients in the better surviving Cluster2 and low SRscores groups had lower mRNAsi. The SRscores and mRNAsi showed a significant positive correlation. Our findings were similar to previous studies in that patients with high mRNAsi had a poorer prognosis. Patients in the better surviving Cluster2 and low SRscores groups had lower mRNAsi. The interventional risk score and mRNAsi showed a significant positive correlation. Studies have shown that the mRNAsi, such as Wnt, Nuclear factor-κB (NF-κB), Janus kinase/signal transducers and activators of transcription (JAK-STAT), Phosphatidylinositol-3-kinase (PI3K)/AKT/mammalian target of rapamycin (mTOR) and other signaling pathways can regulate the growth of CSCs [58]. We found that the high SRscores group was significantly enriched in these pathways. In conclusion, we suggest that the SRscores can reflect the characteristics of CSCs.

We did transcriptional and single-cell analyses. Stem cells had a considerably higher SRscores. Stem cells had much higher gene expression than other cells. Our single-cell data only examined HCC tumor tissues, therefore these genes may be substantially expressed in CSCs. Transcription factors influence tumor stem cell biology, according to several studies. Bioinformatic study showed that tumor stem cells activated TCF3, SMARCA4, TFF3, etc. (Fig. 7D, E). Joint transcriptome data analysis suggested these transcription factors regulate stemness risk-related genes. Single-cell sequencing from hepatocellular cancer stem cells did not evaluate these genes. We can improve risk-scoring models and CSC treatment by studying the distribution and expression of these genes in CSCs. We also have not investigated the detailed regulatory relationships between transcription factors and these genes, which would allow us to investigate the specific networks of stemness genes that regulate tumorigenesis and development.

Eight genes were utilized to generate a stemness-related risk score.LPCAT1 (Lysophosphatidylcholine Acyltransferase 1) is a gene that codes for a protein that is essential for phospholipid metabolism.LPCAT1 is crucial to the development of diverse tumors and may function as a potential prognostic marker. [59–62]. Uehara et al. found that LPCAT1 expression was significantly higher in gastric cancer compared to paraneoplastic tissue [63]. It has been shown that LPCAT1 can promote endometrial tumor cell growth and stemness by affecting the TGF-β signaling pathway [61].In HCC, LPCAT1 is an oncogene that promotes the growth and metastasis of liver cancer cells [64]. Using a rat model of hepatocellular carcinoma, we found that LPCAT1 expression was substantially higher in tumor tissues compared to normal tissues. In addition, silencing LPCAT1 inhibited the stemness of hepatocellular carcinoma cells.

In conclusion, stemness-related subtypes and risk scores were developed. We also systematically described their associations with TME immune cell infiltration, drug sensitivity, and TMB. These results demonstrate different subtypes and high and low-risk score groups in HCC patients. These stemness-related genes' interaction is vital in tumor development and treatment. The SRscores may provide new ideas for subsequent assessment of patient survival, guiding individualized treatment and improving the effectiveness of immunotherapy.

Nonetheless, our investigation inevitably has some limitations. The data in our study were obtained from public databases. Although animal and cellular investigations were conducted for validation, additional clinical validation is required to confirm the practical accuracy. Second, there is a paucity of clinical data validation, and large samples will be required in the future to validate the accuracy of the stemness model in hospitals with which we collaborate. The clinical significance of stemness subtypes and SRscores must be investigated further. In addition, only the role of the core gene LPCAT1 in HCC was validated, and the role and mechanism of stemness-related genes in HCC require further validation of functional experiments of other genes in the model.

### Supplementary Information

**Additional file 1: Figure S1.** (A) Univariate Cox regression analysis of 27 stemness-associated genes. (B) Interactions among 11 stemness-related genes. The thickness of the line indicates the strength of the association. The pink color indicates a positive correlation, and the blue color indicates a negative correlation. (C-D) Waterfall plots showing the distribution of somatic mutations in the highest mutation frequency genes in different subtypes. (E–F) Bayesian NMF identification of mutation markers in different subtypes. The middle and lower plots show the relative proportions of the total number of mutations and mutation types. (G) Interactions between the eight genes that comprise the SRscores. Connected lines represent the presence of interactions, and the line's thickness indicates the association's strength; pink represents a positive correlation, and blue represents a negative correlation. (H) Correlation analysis between the eight genes constituting the SRscores. (I) Enrichment analysis of GSVA pathway between high and low SRscores groups. (J) Differential mRNA expression between HCC and normal tissues for eight genes of the SRscores.

**Additional file 2: Figure S2** (A) The relative abundance of each immune cell subpopulation between the high and low SRscores groups. (B) Analysis of immune checkpoint expression between the high and low SRscores groups. (C) Correlation analysis of 8 genes of the SRscores and immune cell subpopulations. (D) Activation and repression of eight genes of the SRscores with tumor-associated phenotypes and pathways. (E–F) Sensitivity analysis of eight genes of the SRscores to certain antitumor drugs and non-immunotherapeutic agents. (G) Differences in tumor-associated signaling pathways between the SRscores and low stem risk score groups, *p < 0.05; **p < 0.01; ***p < 0. 001.

**Additional file 3: Figure S3.** (A) PCA downscaled analysis. (B) Single-cell clustering analysis and visualization using UMAP and tSNE. (C) 3000 high variant genes selection and top 10 high variant genes display. (D-E) Single-cell subgroup annotation display (UMAP and tSNE). (F) SRscores in single cell subpopulation cells (tSNE) is displayed. (G) Enrichment of the eight genes that make up the SRscores in each cell subpopulation. (H) Expression of LPCAT1 and NDRG1 genes in various subpopulations of single cells is shown.

**Additional file 4: Table S1.** Stemness-related gene sets.

**Additional file 5: Table S2.** Stemness-related genes.

## Data Availability

This study analyzed publicly available datasets. These data can be found in the TCGA, ICGC, and GEO datasets.

## Abbreviations

HCC: Hepatocellular carcinoma
LCSCs: Hepatocellular carcinoma stem cells
SRscores: Stemness-related score
CSCs: Cancer stem cells
CAFs: Cancer-associated fibroblasts
TCGA: The Cancer Genome Atlas
ICGC: The International Cancer Genome Consortium
GEO: The Gene Expression Omnibus database
MSigDB: The Molecular Marker Database
OS: Overall survival
CNV: Copy number variation
km: K-means
TME: Tumor microenvironments
TIDE: The Tumor Immune Dysfunction and Exclusion
DEGs: Differentially expressed genes
NC: Negative control
PEI: Polyethylenimine
MDSCs: Myeloid-derived suppressor cells
HER2: Human epidermal growth factor receptor 2
VEGF: Vascular growth factor
NF-κB: Nuclear factor-κB
JAK-STAT: Janus kinase/signal transducers and activators of transcription
PI3K: Phosphatidylinositol-3-kinase
mTOR: Mammalian target of rapamycin
LPCAT1: Lysophosphatidylcholine Acyltransferase 1
