# Supplementary figures and images for: Deep dissection of stemness-related hierarchies in hepatocellular carcinoma
Source: J Transl Med. 2023 Sep 16;21:631. doi: 10.1186/s12967-023-04425-8 (PMC10505333; doi:10.1186/s12967-023-04425-8)

A

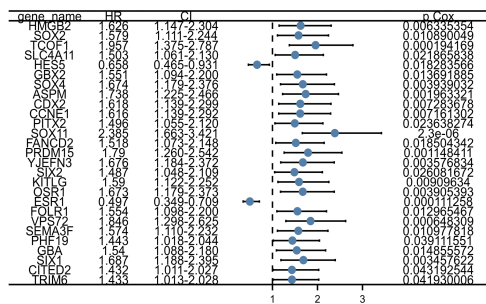

B

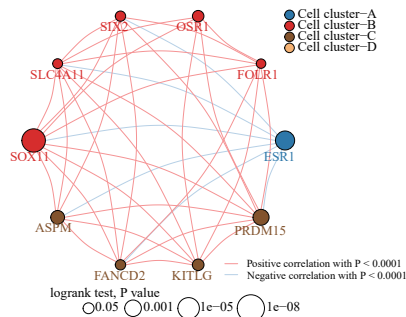

C

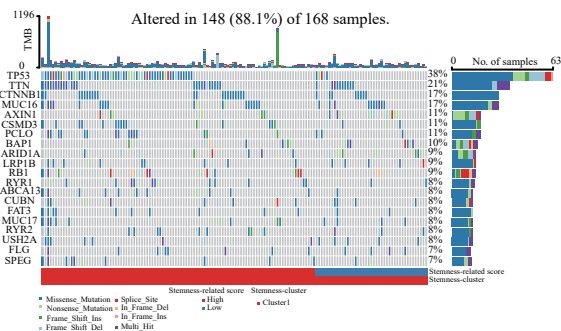

D

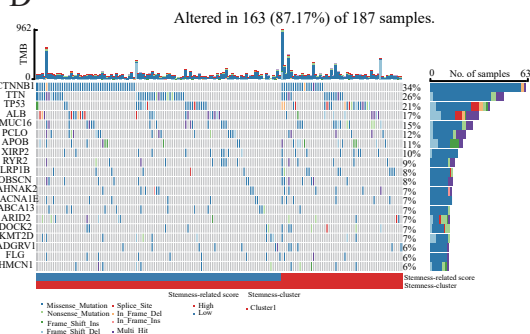

E

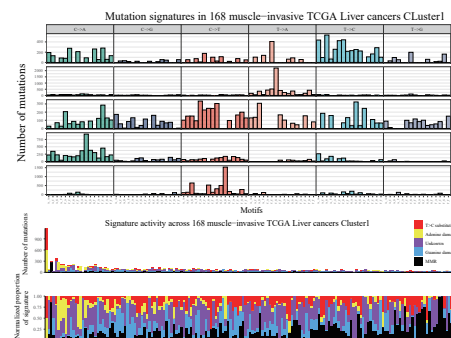

F

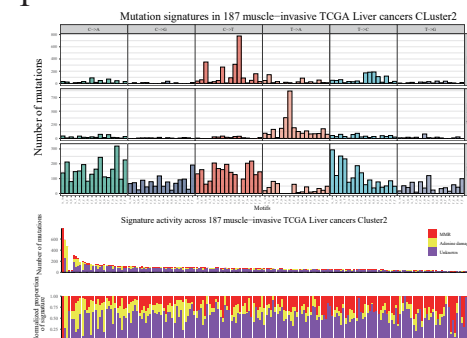

G

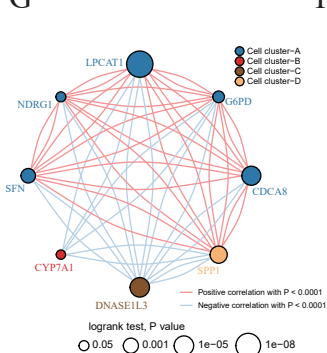

H

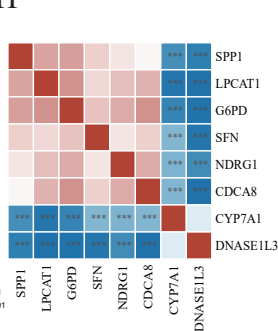

I

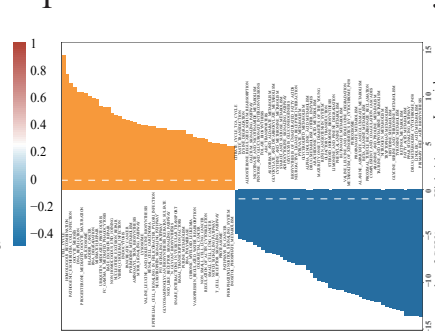

Supplement: Supplementary file 1 — Additional file 1: Figure S1. (A) Univariate Cox regression analysis of 27 stemness-associated genes. (B) Interactions among 11 stemness-related genes. The thickness of the line indicates the strength of the association. The pink color indicates a positive correlation, and the blue color indicates a negative correlation. (C-D) Waterfall plots showing the distribution of somatic mutations in the highest mutation frequency genes in different subtypes. (E–F) Bayesian NMF identification of mutation markers in different subtypes. The middle and lower plots show the relative proportions of the total number of mutations and mutation types. (G) Interactions between the eight genes that comprise the SRscores. Connected lines represent the presence of interactions, and the line's thickness indicates the association's strength; pink represents a positive correlation, and blue represents a negative correlation. (H) Correlation analysis between the eight genes constituting the SRscores. (I) Enrichment analysis of GSVA pathway between high and low SRscores groups. (J) Differential mRNA expression between HCC and normal tissues for eight genes of the SRscores. [file 12967_2023_4425_MOESM1_ESM.pdf]
