# Supplementary material for: Deep dissection of stemness-related hierarchies in hepatocellular carcinoma
Source: J Transl Med. 2023 Sep 16;21:631. doi: 10.1186/s12967-023-04425-8 (PMC10505333; doi:10.1186/s12967-023-04425-8)
Supplement: Supplementary file 4 — Additional file 4: Table S1. Stemness-related gene sets. [file 12967_2023_4425_MOESM4_ESM.docx]

| Stem cell function related pathways | PathwayID | Gene Count |
| --- | --- | --- |
| GO: Somatic Stem Cell Population Maintenance | GO: 0035019 | 45 |
| GO: Negative Regulation of Stem Cell Differentiation | GO: 2000737 | 25 |
| GO: Stem Cell Proliferation | GO: 0072089 | 81 |
| GO: Hematopoietic Stem Cell Differentiation | GO: 0060218 | 30 |
| GO: Negative Regulation of Stem Cell Proliferation | GO: 2000647 | 10 |
| GO: Stem Cell Division | GO: 0017145 | 30 |
| GO: Hematopoietic Stem Cell Proliferation | GO: 0071425 | 29 |
| GO: Positive Regulation of Stem Cell Differentiation | GO: 2000738 | 20 |
| GO: Regulation of Stem Cell Population Maintenance | GO: 2000036 | 38 |
| GO: Neuronal Stem Cell Population Maintenance | GO: 0097150 | 24 |
| GO: Regulation of Stem Cell Proliferation | GO: 0072091 | 54 |
| GO: Somatic Stem Cell Division | GO: 0048103 | 11 |
| GO: Stem Cell Differentiation | GO: 0048863 | 235 |
| GO: Positive Regulation of Stem Cell Proliferation | GO: 2000648 | 33 |
| GO: Regulation of Stem Cell Differentiation | GO: 2000736 | 65 |
| GO: Hematopoietic Stem Cell Migration | GO: 0035701 | 9 |
| GO: Stem Cell Fate Commitment | GO: 0048865 | 8 |
| GO: Mesenchymal Stem Cell Maintenance Involved In Nephron Morphogenesis | GO: 0072038 | 6 |
| GO: Mesenchymal Stem Cell Differentiation | GO: 0072497 | 13 |
| GO: Mesenchymal Stem Cell Proliferation | GO: 0097168 | 8 |
| GO: Asymmetric Stem Cell Division | GO: 0098722 | 2 |
| GO: egulation of Hematopoietic Stem Cell Proliferation | GO: 1902033 | 13 |
| GO: ositive Regulation of Hematopoietic Stem Cell Proliferation | GO: 1902035 | 7 |
| GO: egative Regulation of Stem Cell Population Maintenance | GO: 1902455 | 8 |
| GO: ositive Regulation of Stem Cell Population Maintenance | GO: 1902459 | 10 |
| GO: egulation of Somatic Stem Cell Population Maintenance | GO: 1904672 | 10 |
| GO: Negative Regulation of Somatic Stem Cell Population Maintenance | GO: 1904673 | 5 |
| GO: Regulation of Stem Cell Division | GO: 2000035 | 13 |
| GO: Regulation of Mesenchymal Stem Cell Differentiation | GO: 2000739 | 8 |
| Total |  | 850 |
