# Supplementary material for: Deep dissection of stemness-related hierarchies in hepatocellular carcinoma
Source: J Transl Med. 2023 Sep 16;21:631. doi: 10.1186/s12967-023-04425-8 (PMC10505333; doi:10.1186/s12967-023-04425-8)
Supplement: Supplementary file 5 — Additional file 5: Table S2. Stemness-related genes. [file 12967_2023_4425_MOESM5_ESM.docx]

| **Stemness related genes** | | | | | | | | |
| --- | --- | --- | --- | --- | --- | --- | --- | --- |
| ASCL2 | WNT9B | GBA | TBX3 | FUT10 | WDR43 | EOMES | MSI2 | SEMA4F |
| BCL9 | YAP1 | GJA1 | TERT | FZD7 | ZC3H13 | EPOP | MSX1 | SEMA4G |
| BMP7 | ZFP36L2 | HMGA2 | THPO | GNL3 | ZNF322 | ERBB4 | MSX2 | SEMA5A |
| CDX2 | ZHX2 | HMGB2 | TRIM71 | ING2 | ZNF706 | ESR1 | MTF2 | SEMA5B |
| CUL4A | CDK12 | KAT7 | VEGFC | NAP1L2 | ASPM | FAM172A | NOLC1 | SEMA6A |
| FGF10 | CDK13 | KDF1 | WNT1 | NCOA3 | CDH2 | FBXL17 | NRG1 | SEMA6B |
| GATA2 | ESRRB | KDM1A | WNT10B | PAFAH1B1 | DLL1 | FGF19 | NRP1 | SEMA6C |
| HES1 | GSK3B | KITLG | WNT2B | PRDM15 | FANCC | FGFR2 | NRP2 | SEMA6D |
| HNF1B | H1-8 | LTBP3 | WNT3 | SMYD5 | FANCD2 | FN1 | NRTN | SEMA7A |
| KIT | HES5 | MECOM | WNT5A | TGFB2 | FOXO1 | FOLR1 | NSUN2 | SETD2 |
| KLF10 | HNRNPU | MIR16-1 | WNT7B | TIAL1 | FOXO3 | FOXA1 | OCIAD1 | SETD6 |
| LBH | HSPA9 | MIR221 | YJEFN3 | WWTR1 | HOOK3 | FOXC2 | OSR1 | SHC4 |
| LDB1 | JAG1 | MIR222 | ZFP36L1 | ZBTB16 | IGF2BP1 | FOXO4 | OTUD5 | SHH |
| LDB2 | N4BP2L2 | MIR29B1 | ZNRF3 | DHX36 | MCPH1 | FRZB | PDCD6 | SIX1 |
| LIG4 | NELFB | MIR320A | ABL1 | GATA4 | MMP24 | FZD1 | PDGFRA | SLC4A11 |
| LRP5 | NFE2L2 | MIR320B1 | BATF | GATA6 | PCM1 | GBX2 | PDX1 | SMAD4 |
| MIR145 | NOTCH1 | MIR320B2 | CDK6 | MTCH2 | PROX1 | GDNF | PEF1 | SOX10 |
| MYC | PRICKLE1 | MIR320C1 | ERCC2 | NKX2-5 | PRRX1 | GPM6A | PHACTR4 | SOX21 |
| NANOG | STAT3 | MIR320C2 | EXT1 | NUDT21 | SRRT | GSC | PHF19 | SOX8 |
| NOG | TCF15 | MIR320D1 | FOXC1 | PTN | SS18 | HAND2 | PHF5A | TAPT1 |
| NR2E1 | TRIM6 | MIR320D2 | HOXB4 | PWP1 | ACVR1 | HESX1 | PHOX2B | TBX1 |
| PAX2 | WNT3A | MIR320E | ITCH | RBM24 | ALDH1A2 | HIF1A | PITX2 | TCOF1 |
| PAX8 | YTHDF2 | NES | LMBR1L | SOX5 | ALX1 | HOXA7 | PSMD11 | TEAD2 |
| POU5F1 | ABCB1 | NF2 | MEOX1 | SOX6 | ANXA6 | HTR2B | PUM1 | TWIST1 |
| RAF1 | ACE | NKAP | METTL3 | TACSTD2 | BMP4 | ISL1 | RADIL | WNT10A |
| RBPJ | AGO3 | OVOL1 | MLLT3 | TBX5 | BMPR1A | JARID2 | RDH10 | WNT8A |
| REST | ARIH2 | OVOL2 | OSM | CNOT1 | CDC42 | KBTBD8 | RET | ZNF281 |
| SFRP1 | ATXN1L | PDCD2 | PRKDC | CNOT2 | CITED2 | KDM4C | RUNX2 | BCL11B |
| SIX2 | CCNE1 | PIM1 | PUS7 | CNOT3 | CORO1C | KLHL12 | SEMA3A | CCR2 |
| SKI | CD34 | PTPRC | SETD1A | ELAVL1 | CTNNB1 | LAMA5 | SEMA3B | GAS6 |
| SOX2 | CITED1 | RNF43 | SP7 | KAT2A | CYP26C1 | LIF | SEMA3C | GPLD1 |
| SOX4 | CTC1 | RUNX1 | SRF | KDM2B | DNMT3L | LIN28A | SEMA3D | JAM2 |
| SOX9 | EIF2AK2 | SART3 | TAL1 | KDM3A | EDN1 | LRP6 | SEMA3E | JAM3 |
| SPI1 | EPCAM | SFRP2 | TP53 | LOXL2 | EDN3 | MAPK1 | SEMA3F |  |
| TAF5L | ETV6 | SIRT6 | UFL1 | NODAL | EDNRA | MAPK3 | SEMA3G |  |
| TAF6L | FBLN1 | SNAI2 | XRCC5 | PRDM14 | EDNRB | MEF2C | SEMA4A |  |
| TP63 | FERMT1 | SOX11 | CDK2AP2 | SAV1 | EEF1AKMT4-ECE2 | METTL5 | SEMA4B |  |
| VPS72 | FERMT2 | SOX17 | CUL3 | SMO | EFNB1 | MIR146A | SEMA4C |  |
| WNT7A | FGF2 | SOX18 | EVI2B | TEAD4 | ELL3 | MIR346 | SEMA4D |  |
