# Supplementary material for: Deep dissection of stemness-related hierarchies in hepatocellular carcinoma
Source: J Transl Med. 2023 Sep 16;21:631. doi: 10.1186/s12967-023-04425-8 (PMC10505333; doi:10.1186/s12967-023-04425-8)
Supplement: Supplementary file 2 — Additional file 2: Figure S2 (A) The relative abundance of each immune cell subpopulation between the high and low SRscores groups. (B) Analysis of immune checkpoint expression between the high and low SRscores groups. (C) Correlation analysis of 8 genes of the SRscores and immune cell subpopulations. (D) Activation and repression of eight genes of the SRscores with tumor-associated phenotypes and pathways. (E–F) Sensitivity analysis of eight genes of the SRscores to certain antitumor drugs and non-immunotherapeutic agents. (G) Differences in tumor-associated signaling pathways between the SRscores and low stem risk score groups, *p < 0.05; **p < 0.01; ***p < 0. 001. [file 12967_2023_4425_MOESM2_ESM.pdf]

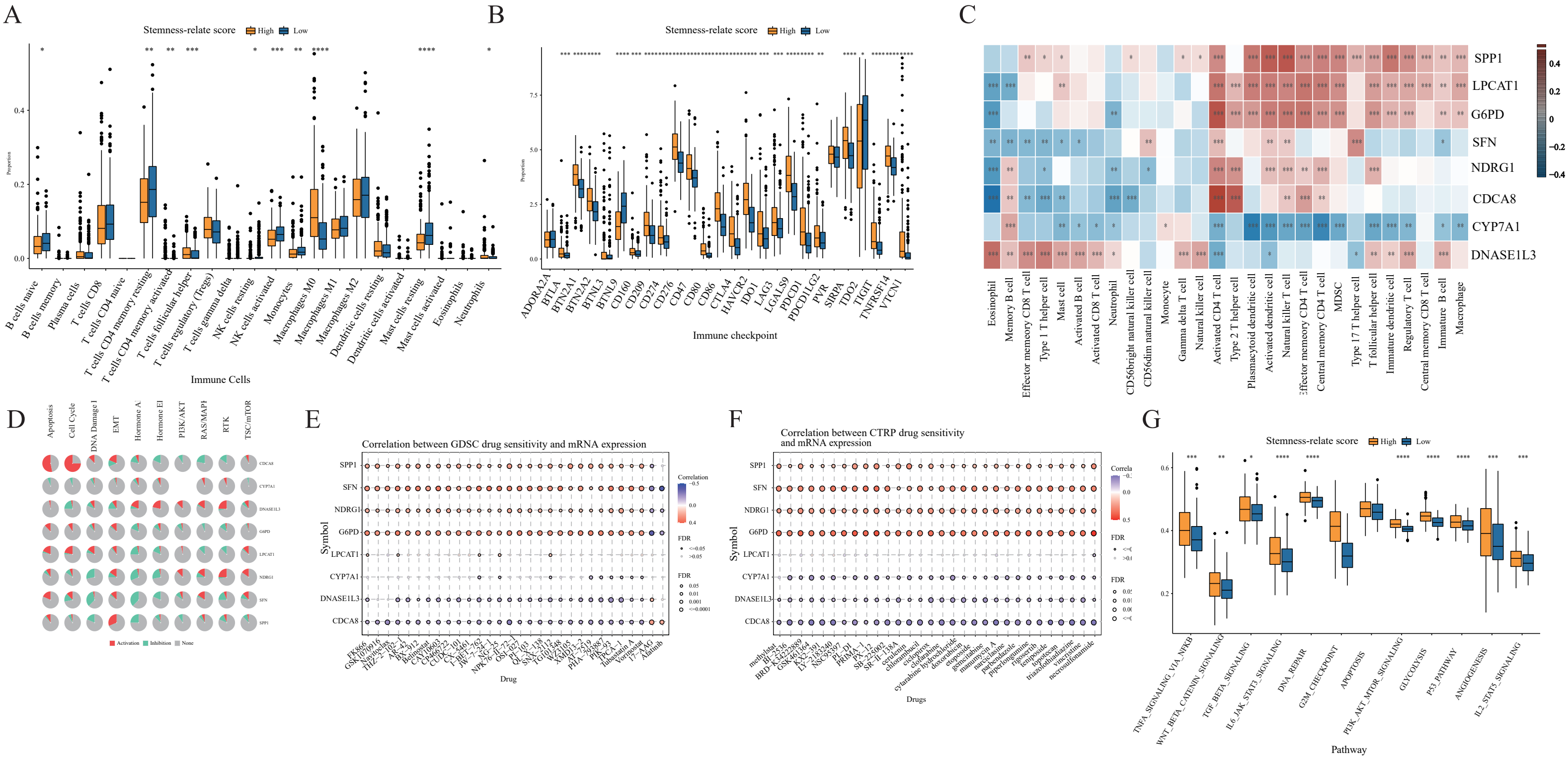

A
Stemness-relate score High Low
Proportion
Immune Cells
B cells naive
B cells memory
Plasma cells
T cells CD8
T cells CD4 naive
T cells CD4 memory resting
T cells CD4 memory activated
T cells follicular helper
T cells regulatory (Tregs)
T cells gamma delta
NK cells resting
NK cells activated
Monocytes
Macrophages M0
Macrophages M1
Macrophages M2
Dendritic cells resting
Dendritic cells activated
Mast cells resting
Mast cells activated
Eosinophils
Neutrophils
B
Stemness-relate score High Low
Proportion
Immune checkpoint
ADORA2A
BTLA
BTN2A1
BTN2A2
BTNL3
BTNL9
CD160
CD209
CD274
CD276
CD47
CD80
CD86
CTLA4
HAVCR2
IDO1
LAG3
LGALS9
PDCD1
PDCD1LG2
PVR
SIRPA
TDO2
TIGIT
TNFRSF14
VTCN1
C
SPP1
LPCAT1
G6PD
SFN
NDRG1
CDCA8
CYP7A1
DNASE1L3
Eosinophil
Memory B cell
Effector memeory CD8 T cell
Type 1 T helper cell
Mast cell
Activated B cell
Activated CD8 T cell
Neutrophil
CD56bright natural killer cell
CD56dim natural killer cell
Monocyte
Gamma delta T cell
Natural killer cell
Activated CD4 T cell
Type 2 T helper cell
Plasmacytoid dendritic cell
Activated dendritic cell
Natural killer T cell
Effector memory CD4 T cell
Central memory CD4 T cell
MDSC
Type 17 T helper cell
T follicular helper cell
Immature dendritic cell
Regulatory T cell
Central memory CD8 T cell
Immature B cell
Macrophage
D
Apoptosis
Cell Cycle
DNA Damage
EMT
Hormone AR
Hormone ER
PI3K/AKT
RAS/MAPK
RTK
TSC/mTOR
CDCA8
CYP7A1
DNASE1L3
G6PD
LPCAT1
NDRG1
SFN
SPP1
Activation Inhibition None
E
Correlation between GDSC drug sensitivity and mRNA expression
Symbol
Drug
Correlation
FDR
<=0.05
>0.05
F
Correlation between CTRP drug sensitivity and mRNA expression
Symbol
Drugs
G
Stemness-relate score High Low
Pathway
TNFA_SIGNALING_VIA_NFKB
WNT_BETA_CATENIN_SIGNALING
TGF_BETA_SIGNALING
IL6_JAK_STAT3_SIGNALING
DNA_REPAIR
G2M_CHECKPOINT
APOPTOSIS
PI3K_AKT_MTOR_SIGNALING
GLYCOLYSIS
P53_PATHWAY
ANGIOGENESIS
IL2_STAT5_SIGNALING
