# Supplementary material for: Deep dissection of stemness-related hierarchies in hepatocellular carcinoma
Source: J Transl Med. 2023 Sep 16;21:631. doi: 10.1186/s12967-023-04425-8 (PMC10505333; doi:10.1186/s12967-023-04425-8)
Supplement: Supplementary file 3 — Additional file 3: Figure S3. (A) PCA downscaled analysis. (B) Single-cell clustering analysis and visualization using UMAP and tSNE. (C) 3000 high variant genes selection and top 10 high variant genes display. (D-E) Single-cell subgroup annotation display (UMAP and tSNE). (F) SRscores in single cell subpopulation cells (tSNE) is displayed. (G) Enrichment of the eight genes that make up the SRscores in each cell subpopulation. (H) Expression of LPCAT1 and NDRG1 genes in various subpopulations of single cells is shown. [file 12967_2023_4425_MOESM3_ESM.pdf]

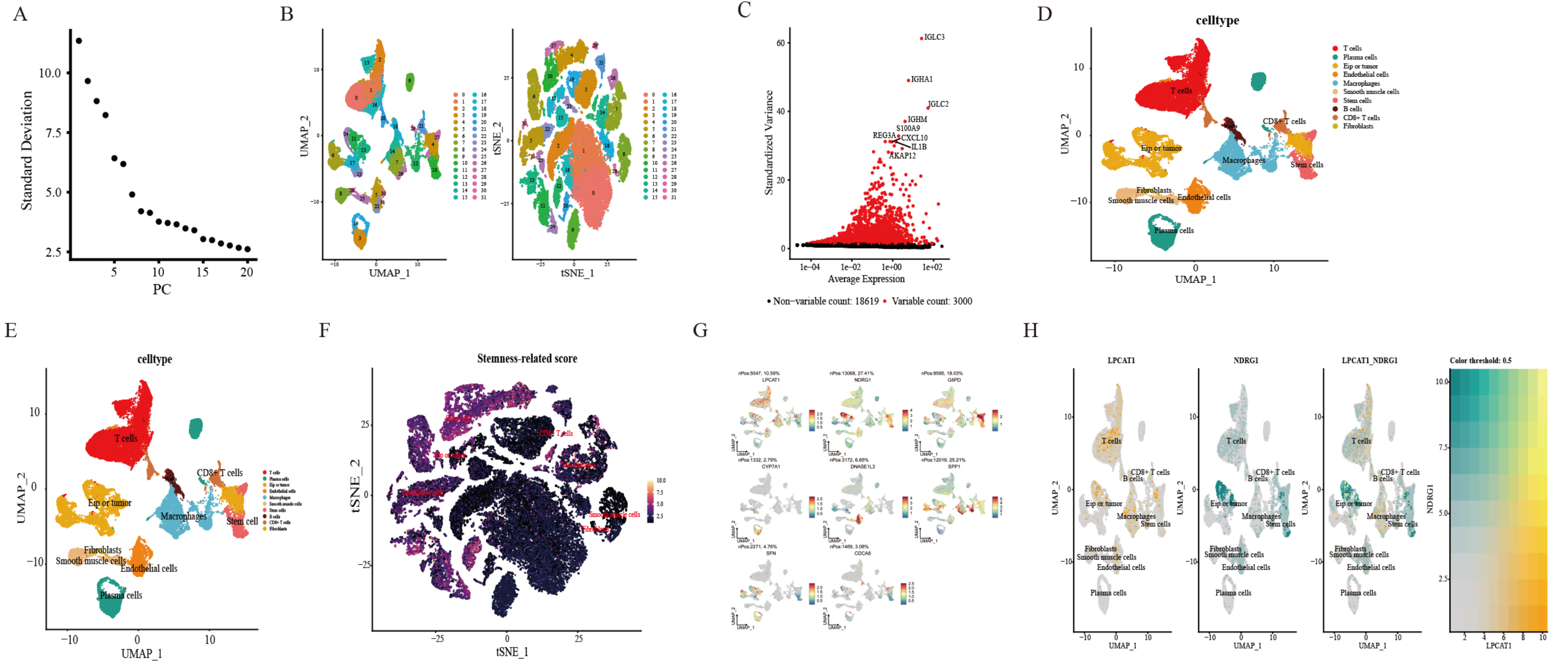

A
Standard Deviation
PC
B
UMAP_2
UMAP_1
tSNE_2
tSNE_1
C
Standardized Variance
Average Expression
IGLC3
IGHA1
IGLC2
IGHM
S100A9
REG3A
CXCL10
IL1B
AKAP12
• Non−variable count: 18619 • Variable count: 3000
D
celltype
T cells
Plasma cells
Eip or tumor
Endothelial cells
Macrophages
Smooth muscle cells
Stem cells
B cells
CD8+ T cells
Fibroblasts
UMAP_2
UMAP_1
E
celltype
F
Stemness-related score
tSNE_2
tSNE_1
G
nPos:5047, 10.59%
LPCAT1
nPos:13068, 27.41%
NDRG1
nPos:8595, 18.03%
G6PD
nPos:1332, 2.79%
CYP7A1
nPos:3172, 6.65%
DNASE1L3
nPos:12019, 25.21%
SPP1
nPos:2271, 4.76%
SFN
nPos:1469, 3.08%
CDCA8
H
LPCAT1
NDRG1
LPCAT1_NDRG1
Color threshold: 0.5
NDRG1
LPCAT1
